# Neurological Examination Techniques of Speech in *Bahasa Malaysia* for Adults: Simple Approach Practiced in Hospital Universiti Sains Malaysia

**DOI:** 10.21315/mjms2020.27.6.14

**Published:** 2020-12-29

**Authors:** Abdul Haleem Noorsham, Mohamad Muhaimin Abdullah, Sanihah Abdul Halim, Abdul Rahman Izaini Ghani, Zamzuri Idris, Jafri Malin Abdullah

**Affiliations:** 1 Department of Neurosciences, School of Medical Sciences, Universiti Sains Malaysia, Kelantan, Malaysia; 2 Department of Neurosciences, Hospital Universiti Sains Malaysia, Universiti Sains Malaysia, Kelantan, Malaysia; 3 Brain and Behaviour Cluster, School of Medical Sciences, Universiti Sains Malaysia, Kelantan, Malaysia; 4 Department of Neurosurgery, Hospital Sultanah Nur Zahirah, Terengganu, Malaysia; 5 Department of Neurosurgery, Hospital Sultanah Bahiyah, Kedah, Malaysia; 6 Neurology Unit, Department of Internal Medicine, School of Medical Sciences, Universiti Sains Malaysia, Kelantan, Malaysia

**Keywords:** speech disorders, speech examination, dysphasia, dysarthria, Broca’s dysphasia, Wernicke’s dysphasia, Boston diagnostic aphasia examination, Western aphasia battery-revised, Bahasa Malaysia

## Abstract

There are four classification levels for speech disorders namely dysphonia, dysarthria, dysprosody and dysphasia. In general, speech examination mainly focuses on three main components that are spontaneous speech, auditory comprehension, and oral motor examination. Quick bedside assessment on speech in *Bahasa Malaysia* is essential to assist the speech language therapist (SLT) and other physicians to determine the disorders. Speech therapy is also essential in monitoring and continuous assessment for patients with speech and language disorders such as dysphasia and dysarthria. Speech clinicians in Hospital Universiti Sains Malaysia (HUSM) have been adapting two most widely used batteries of speech assessment tools namely Western aphasia battery-revised (WAB-R) by Andrew Kertesz and Boston diagnostic aphasia examination (BDAE). These tools have been modified into simple and validated speech assessments in *Bahasa Malaysia*. This video manuscript will demonstrate the use of both tools in performing bedside speech assessment for patients with speech disorders. The *Bahasa Malaysia* speech examination should not be difficult when WAB-R and BDAE speech assessment tools are applied. The aim of this simple approach using the adapted version of BDAE and WAB-R is to assist the clinician to achieve quick and accurate diagnosis with a validated scoring system.

## Introduction

There are 32.7 million people in Malaysia with diverse cultures and sharing different beliefs, customs, values, religions and languages (estimated: 69.1% Malay, 23.0% Chinese, 6.9% Indians and 1% others) (1). A state in East Malaysia, Kelantan, consists of approximately 1.8 million Malay ethnicities (1) who speaks Malay dialect (2). The official language of Malaysia is the Malay language (*Bahasa Melayu* or *Bahasa Malaysia*) whereas English serves as the second language. A communicative speech consists of words arranged according to the rules of grammar and syntax and invested with prosody.

Speech disorders can be classified into four levels according to the site of lesions. Lesion at the lowest level is known as dysphonia due to the disturbance in the production of sounds in the larynx. Dysarthria is defined as a disorder in articulating speech sounds, which is due to the second lowest lesion. The next level of lesion is dysprosodies consisting of scanning speech (cerebellar), plateau speech (basal motor nuclei/Parkinsonian), and stuttering, cluttering and absence of emotional inflections (cerebral). The highest level of lesion is known as dysphasia that is defined as the disturbance in the understanding or expression of words as symbols for communication (3). The two most common types of dysphasia are expressive and receptive dysphasia. Expressive dysphasia or known as Broca’s aphasia is the non-fluent type of aphasia whereby the patient speaks telegraphically, sparsely, and slowly. Furthermore, receptive aphasia is known as Wernicke aphasia or fluent aphasia whereby a lesion happened in the Wernicke area of the brain that is responsible for comprehension of spoken words or written words (3).

Malaysia is known as multi-lingual country hence, speech examination among Malaysians is a huge challenge. The speech language therapist (SLT) in Hospital Universiti Sains Malaysia (HUSM) has been using two modified diagnostic aphasia batteries known as the Boston diagnostic aphasia examination (BDAE) (Appendix 1) (4) and Western aphasia battery-revised (WAB-R) by Andrew Kertesz (Appendix 2) (5). These tools have been used to perform a neurological examination of speech in HUSM among the Malay language speakers in Kelantan. BDAE and WAB-R are the best batteries to achieve an accurate diagnosis of speech disorders in adults. These batteries have been carefully adapted and translated into *Bahasa Malaysia* for the ease of communication and quick bedside assessment.

The goals for the examination are to determine the types of speech disorders, particularly expressive or receptive dysphasia, dysarthria, disorders in reading and writing or other oral motor disorders. The adapted assessment tools in general focus on three main components of speech examinations namely spontaneous speech, auditory comprehension and oral motor examinations. Reading and writing are included under the linguistic component, which is an extension from the three main core areas. The examinations scores are recorded at the end of the assessment and repeated within a certain period of time. Any improvement or deterioration from the first assessment can be monitored by per cases basis.

## Methods

In general, the goal of speech examination in *Bahasa Malaysia* is to classify speech disorders into the types of aphasia namely expressive or receptive, dysarthria or other oral motor discrepancies. This can be achieved by focusing on the three main components of speech assessment including spontaneous speech, auditory comprehension and oral motor examination that includes repetition and naming (3). The fourth component, which is linguistic that includes extensive reading and writing examination, is tested thoroughly in a clinical setting (3). For practical purposes, the video of speech examination in *Bahasa Malaysia* can be viewed together with this article.

The speech examination performed at the bedside or at the clinic is performed using the adapted version of BDAE and WAB-R. Both batteries have been translated into *Bahasa Malaysia* in order for the SLT in HUSM to perform a better assessment. These two batteries are being used worldwide, adapted to various languages and not limited for diagnostic purposes, but also evaluation of language functionality and treatment designed for patients with aphasia (6, 7). Many groups have used these batteries that have been translated into *Bahasa Malaysia* in language assessments and treatment designs (8, 9, 10, 11, 12, 13,14).

## Speech Assessment Typical Components

### Case History

Case history consists of a collection of information about speech difficulties particularly in the areas of language processes. These include difficulty in talking, hearing, vision, swallowing and other important medical conditions that may be related to the speech complaints. The medication history is also important especially the side effects of certain medications. Other histories include family, education level and patient’s language proficiency in all language modalities. Patient’s communication needs, as well as the family’s expectation for therapy, is vital during case history session.

### Spontaneous Speech Examination

The aims of spontaneous speech examination at the bedside are essential to evaluate the number of words spoken, the content and the associations of the sentences produced by the patient. These are essential for the physicians to determine the adequacy of response from the patient. Furthermore, another common method to initiate a response from the patient during spontaneous speech session is to ask the patient to tell a story or describing a picture to the examiner. This will aid the examiner to evaluate the types of conversation produced by the patient. The response was scored according to the contents of the conversation as seen in (Appendix 1) and (Appendix 2).

This first assessment plays important role in determining the fluency of patient’s speech to direct the assessment pathway in further evaluating the speech disorders at the end of the assessment. Patient that is non-fluent in speech but has an appropriate content will be directed to the auditory speech comprehension in view that the patient may have a lesion at the Broca’s region (speech production).

### Auditory Comprehension

Comprehension test consists of word comprehension, commands and complex ideational material. The sentences are verbalised by the examiner and the patient has to pinpoint to one of four visual stimuli indicating the correct sentence. The patient also has to perform a spoken command in which the patient has to answer the questions correctly. Finally, a short story will be read to the patient and the patient needs to answer questions related to the story (Appendix 1) (Appendix 2). The aim of this test is to capture either the patient has impaired comprehension in understanding grammar and syntax, words in relation to other words or difficulty with semantics and understanding individual words.

### Oral Expression

Oral expression assesses two major areas, which are repetition and naming. These two components are important to distinguish the patient either anomic or conductive aphasia whereby the lesion is at dominant parietal or arcuate fasciculus, respectively. The patient needs to recite sentences under automatised sequences, repeat single, sentences, naming objects and basic symbols as shown by the examiner (Appendix 1) (Appendix 2). The aim of oral motor expression is to determine any deficit in articulation that is responsive to certain muscles involved in producing speech.

### Reading

Reading test is performed to test the patient’s ability to comprehend written language symbols by reading. Written language is perceived by the visual system and the information conveyed to the perisylvian language centers. Patient is also asked to read basic symbol using visual cues and the patient has to choose appropriate words related to the pictures being shown and reading comprehension (Appendix 1) (Appendix 2). The inability of the patient to read (alexia) reflects a dysfunction to the language centers or interruptions of the connections with the visual system.

### Writing

Patients who are aphasic in speech are also aphasic in writing, however, writing ability may be preserved in patients with dysarthria or verbal apraxia. The ability of the patients to perform mechanical writing is assessed before writing words, repeating the spoken objects and drawing the pictures shown by the examiner. The test was ended by requesting the patient to write a story based on a picture and the content is evaluated (Appendix 1) (Appendix 2). The areas of writing such as mechanical, grammar, syntax and contents are scored accordingly.

### Assessment Tools

The accurate and quick diagnosis from bedside to full speech examination to determine the types of aphasia is important in confirming the localisation of cortical to oral motor lesions. This will assist the clinician in designing the best effective treatment program for the patients. At present, many assessment tools are available to perform speech examination, which can be divided into formal and informal (Table 1). This paper summarises the commonly used assessment tools by HUSM speech clinicians in clinic and bedside settings. The tools are divided to classification and non-classification test of aphasia (Table 1) (15). The classification test for aphasia that is commonly used and adapted to multi-languages are WAB-R and BDAE whereby the non-classification test for aphasia are Minnesota test for differential diagnosis of aphasia (MTDDA) (19), Porch index of communicative ability (PICA) (20), American speech and hearing association functional assessment of communication skills (ASHA FACS) (21), and communication abilities in daily living (C-ADL) (22). However, for formal screening language test, the Frenchay aphasia screening test (FAST) (18) and the language screening test (LAST) (16) has been validated to be used in emergency setting. The MTDDA and PICA tests are used for prognostic purpose whereby the ASHA FACS aims towards functional assessment on the effect of aphasia on the patient’s daily activities (21). The most commonly used formal assessment tool by speech-language therapist in HUSM is the BDAE. This tool has been adapted into *Bahasa Malaysia* and has been used since 2012 (Appendix 1). The adaptation of these two batteries to *Bahasa Malaysia* reflects that *Bahasa Malaysia* is the main language while Malay dialect is spoken by the majority of Kelantanese people (2). The northeastern region of Peninsular Malaysia has the largest Malay language speakers due the location that is at Malaysia-Thailand border (23). Furthermore, the assessment tool is translated into Malay language since it is part of national education system and is also used for formal duty at school and working place (24). This adapted version has been used for many years in HUSM and its reliability and validity correspond to the original adaptation of BDAE, which has been used worldwide as aphasia test. The clinicians in HUSM also adapted the WAB-R, which is one of the popular aphasia tests used worldwide (Appendix 2).

Video on speech examination in *Bahasa Malaysia* accompanies this manuscript at https://youtu.be/uyVVn4X-cSo

## Outcome of the Assessment

Through the formal assessment using the adapted version, the examination of speech in *Bahasa Malaysia* aims to examine linguistic skills such as information content, fluency, auditory comprehension, repetition, naming and word finding, reading and writing as well as non-linguistic skills such as drawing, calculation, block design, and praxis of adults with aphasia. Both batteries have different scoring systems whereby the BDAE uses percentile rank scoring system (3) whereas WAB-R uses point scoring system (4). The outcome of both assessment tools is to achieve an accurate diagnosis for aphasia classification, to evaluate the severity of aphasia, and to determine the adult’s language functionality with aphasia for rehabilitation and treatment programme.

## Conclusion and Future Studies Directions

In this paper, the simple approach to examine *Bahasa Malaysia* speech using the adapted version of worldwide aphasia tests namely BDAE and WAB-R was described. These two batteries have been validated for reliability and validity to be used in many other languages apart from *Bahasa Malaysia*. The aims of this simple approach in examining speech in Malay speaking Malaysian population are to assist the speech clinicians and the non-speech clinicians to determine the types of aphasia in patients presented with acute setting or in the clinical setting.

## Figures and Tables

**Table 1:** List of assessment tools for classification and non-classification test of aphasia

Formal assessments	Informal assessment
**Classification test**	
WAB – Western aphasia battery (5)	Bedside Neurological Speech Examination
BDAE – Boston diagnostic aphasia examination (4)	–
ANELT – Amsterdam-Nijmegen everyday language test (17)	–
**Formal screening tools**	
FAST – Frenchay aphasia screening test (18)	
The language screening test (LAST) (16)	–
**Non-classification test**	
MTDDA – Minnesota test for differential diagnosis of aphasia (19)	–
PICA – Porch index of communicative ability (20)	–
American speech and hearing association functional assessment of communication skills (ASHA FACS) (21)	–
Communication abilities in daily living (C-ADL) (22)	–
